# Iron deficiency is associated with Hypothyroxinemia and Hypotriiodothyroninemia in the Spanish general adult population: Di@bet.es study

**DOI:** 10.1038/s41598-018-24352-9

**Published:** 2018-04-26

**Authors:** Cristina Maldonado-Araque, Sergio Valdés, Ana Lago-Sampedro, Juan Antonio Lillo-Muñoz, Eduardo Garcia-Fuentes, Vidal Perez-Valero, Carolina Gutierrez-Repiso, Albert Goday, Ines Urrutia, Laura Peláez, Alfonso Calle-Pascual, Luis Castaño, Contxa Castell, Elias Delgado, Edelmiro Menendez, Josep Franch-Nadal, Sonia Gaztambide, Joan Girbés, Emilio Ortega, Joan Vendrell, Matilde R. Chacón, Felipe J. Chaves, Federico Soriguer, Gemma Rojo-Martínez

**Affiliations:** 1 Department of Endocrinology and Nutrition, Hospital Regional Universitario de Málaga, IBIMA, Málaga, Spain; 2 Centro de Investigación Biomédica en Red de Diabetes y Enfermedades Metabólicas Asociadas (CIBERDEM), Instituto de Salud Carlos III, Madrid, Spain; 3 UGC de Laboratorio (Bioquímica). Hospital Regional Universitario de Málaga, Málaga, Spain; 4 UGC de Aparato Digestivo. Hospital Universitario Virgen de la Victoria, IBIMA, Málaga, Spain; 5 CIBER de Fisiopatología de la Obesidad y Nutrición (CIBEROBN), Instituto de Salud Carlos III, Madrid, Spain; 6 Department of Endocrinology and Nutrition, Hospital del Mar, Barcelona, Spain; 7 Hospital Universitario Cruces, BioCruces, UPV/EHU, Barakaldo, Spain; 8 Department of Endocrinology and Nutrition, Hospital Universitario S. Carlos de Madrid, Madrid, Spain; 9 Public Health Agency of Catalonia, Department of Health, Barcelona, Spain; 10 Endocrinology and Nutrition Service, Hospital Universitario Central de Asturias, Oviedo, Spain; 11 EAP Raval Sud, Institut Català de la Salut, Red GEDAPS, Primary Care, Unitat de Suport a la Recerca (IDIAP – Fundació Jordi Gol), Barcelona, Spain; 12 Department of Endocrinology and Nutrition, Hospital Universitario Cruces – UPV-EHU, Baracaldo, Barcelona, Spain; 13 Diabetes Unit, Hospital Arnau de Vilanova, Valencia, Spain; 14 Institut d’Investigacions Biomèdiques August Pi i Sunyer (IDIBAPS), Hospital Clínic de Barcelona, Barcelona, Spain; 15 Department of Endocrinology and Nutrition, Hospital Universitario Joan XXIII, Institut d’Investigacions Sanitaries Pere Virgili, Tarragona, Spain; 16 Genomic Studies and Genetic Diagnosis Unit, Fundación de Investigación del Hospital Clínico de Valencia-INCLIVA, Valencia, Spain; 17 Department of Medicine, University of Oviedo, Hospital Central de Asturias, Oviedo, Spain; 18 Instituto de Investigación Sanitaria del Principado de Asturias (ISPA), Oviedo, Spain; 19 UGC de Endocrinología y Nutrición. Hospital Universitario Virgen de la Victoria. IBIMA, Málaga, Spain; 20 Joan XXIII University Hospital. IISPV., Tarragona, Spain

## Abstract

Previous studies have suggested that iron deficiency (ID) may impair thyroid hormone metabolism, however replication in wide samples of the general adult population has not been performed. We studied 3846 individuals free of thyroid disease, participants in a national, cross sectional, population based study representative of the Spanish adult population. Thyroid stimulating hormone (TSH), free thyroxin (FT4) and free triiodothyronine (FT3) were analyzed by electrochemiluminescence (E170, Roche Diagnostics). Serum ferritin was analyzed by immunochemiluminescence (Architect I2000, Abbott Laboratories). As ferritin levels decreased (>100, 30–100, 15–30, <15 µg/L) the adjusted mean concentrations of FT4 (p < 0.001) and FT3 (p < 0.001) descended, whereas TSH levels remained unchanged (p = 0.451). In multivariate logistic regression models adjusted for age, sex, UI, BMI and smoking status, subjects with ferritin levels <30 µg/L were more likely to present hypothyroxinemia (FT4 < 12.0 pmol/L p5): OR 1.5 [1.1–2.2] p = 0.024, and hypotriiodothyroninemia (FT3 < 3.9 pmol/L p5): OR 1.8 [1.3–2.6] p = 0.001 than the reference category with ferritin ≥30 µg/L. There was no significant heterogeneity of the results between men, pre-menopausal and post-menopausal women or according to the iodine nutrition status. Our results confirm an association between ID and hypothyroxinemia and hypotriiodothyroninemia in the general adult population without changes in TSH.

## Introduction

Iron deficiency (ID) is the most prevalent nutritional deficiency worldwide^1^. It is a major public health problem with important adverse health consequences: It delays psychomotor development and affects cognitive performance in children^2^. It causes damage to immune mechanisms, thereby increasing vulnerability to infectious diseases^3^. ID seriously impairs the ability and physical performance of work in men and women^4^. During pregnancy, ID is associated with multiple adverse outcomes for both mother and child, including increased risk of bleeding, sepsis, maternal mortality, perinatal mortality and low birth weight^5^. In addition to these adverse effects, previous animal^6–8^ and human studies^9,10^ have suggested that ID may impair thyroid hormone metabolism. However, the majority of evidence available concerning the association between ID and thyroid dysfunction remains limited to children and adolescents^11–13^ or pregnant women^14–17^ whereas replication in wide samples of the general adult population has not been carried out. Accordingly, we aimed to test the hypothesis of an association between ID and Hypothyroxinemia and Hypotriiodothyroninemia in a wide population sample, representative of the general adult non-pregnant population of Spain (di@bet.es study).

## Results

### Characteristics of the study sample

Table 1 shows the clinical characteristics of the study sample. A total of 3846 individuals were included in the analysis. This sample was composed of 1793 men (45.9%), 1081 pre-menopausal women (28.1%) and 998 post-menopausal women (25.9%). Mean age of the population was 50.0 ± 17.1 years (range 18–93 years). According to the ferritin concentrations, 13.6% of the population had ferritin levels <15 µg/L, 14.4% had ferritin levels between 15–29 µg/L, 38.3% had ferritin levels between 30–99 µg/L, and 33.7% had ferritin levels ≥100 µg/L, so that 28.0% of the study sample had ID according to the proposed definition (ferritin <30 µg/L). Mean and medium urinary iodine (UI) concentrations were 133.8 ± 80.6 and 116.1 μg/g respectively. Mean and median concentrations of thyroid stimulating hormone (TSH), free thyroxine (FT4) and free triiodothyronine (FT3) were within the expected range, taking into consideration the exclusion criteria for this analysis.

**Table 1:** Clinical characteristics of the study sample (3846 individuals free of thyroid disease).

	Number (%)	Mean ± SD	Median	Range
Age (years)		50.0 ± 17.1		18–93
Men	1767 (45.9)			
Premenopausal women	1081 (28.1)			
Postmenopausal women	998 (25.9)			
Currently smoking	1023 (26.6)			
BMI (kg/m^2^)		28.0 ± 5.1		12.2–61.3
Ferritin (µg/L)				
<15	523 (13.6)			
15–30	554 (14.4)			
30–100	1473 (38.3)			
≥100	1296 (33.7)			
UI (μg/g)		133.8 ± 80.6	116.1	20.0–632.5
TSH (µUI/mL)		2.33 ± 1.36	2.06	0.11–18.50
FT4 (pmol/L)		15.13 ± 2.04	15.05	8.21–26.24
FT3 (pmol/L)		5.01 ± 0.72	4.95	2.59–11.95

BMI: Body mass index. UI: Urinary iodine.

### Prevalence of ID

**Figure 1:**
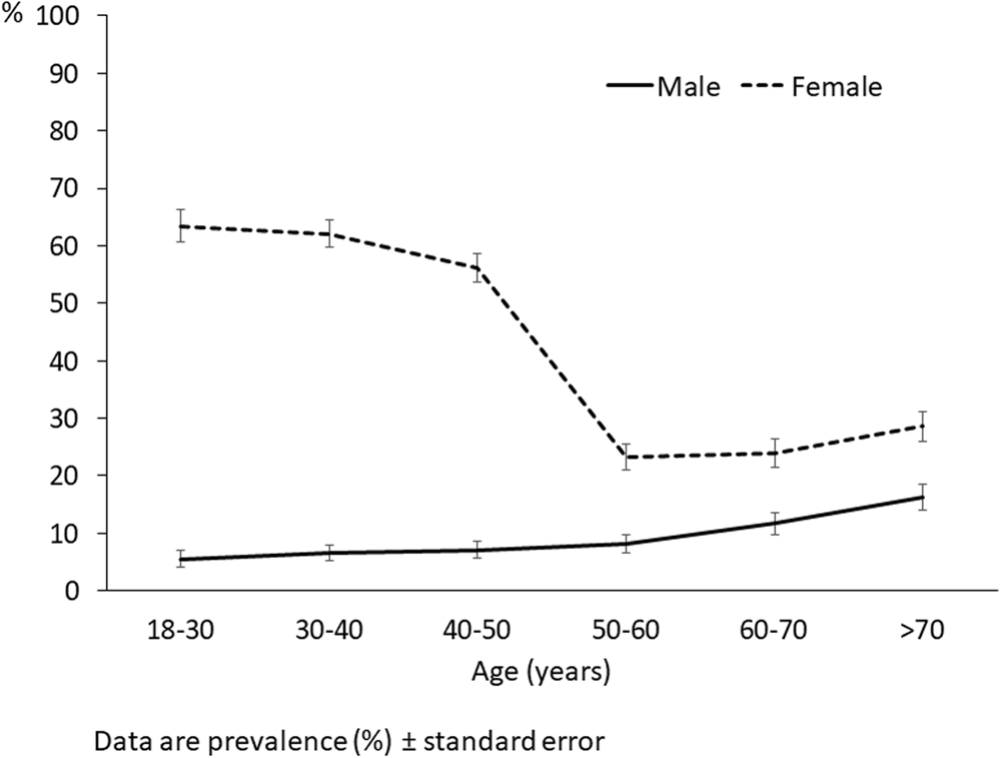
Proportion of the study population with ferritin levels <30 µg/L according to age and gender.

Figure 1 shows the proportion of the population with ID according to gender and age groups. As can be seen, ID was especially prevalent in pre-menopausal women, reaching prevalences of >50% in females in the 18 to 50 year old groups, with a drop in the prevalence in the 50–60 years decade and then a mild increase. Men also showed a slight increase in the prevalence with age. However women had consistently higher prevalence of ID than men in all the age groups.

### Relation between Ferritin levels and thyroid hormones (linear model)

Table 2 shows the mean concentrations of thyroid hormones in the study population according to ferritin levels, in a general linear model adjusted for age, sex, UI, BMI and smoking status. As ferritin levels decreased (>100, 30–100, 15–30, <15 µg/L), the adjusted mean concentrations of FT4 and FT3 descended (p for difference and p for trend for both comparisons <0.001). However, TSH adjusted mean concentrations remained unchanged (p for difference = 0.565, p for trend = 0.451).

**Table 2:** Estimated mean concentrations of TSH, FT4, and FT3 in the study individuals divided according to their Ferritin levels.

	Ferritin (µg/L)				P for difference	P for trend
	<15	15–30	30–100	>100		
TSH (µUI/mL)	1.98 ± 0.07	2.07 ± 0.06	2.02 ± 0.04	2.05 ± 0.04	0.565	0.451
FT4 (pmol/L)	14.83 ± 0.10	15.06 ± 0.09	15.09 ± 0.06	15.33 ± 0.07	<0.001	<0.001
FT3 (pmol/L)	4.91 ± 0.03	4.91 ± 0.03	5.01 ± 0.02	5.11 ± 0.02	<0.001	<0.001

Data are estimated marginal means ± standard errors calculated in a general linear model, adjusted to age, sex, UI, BMI and smoking status.

### Relation between Ferritin levels and thyroid hormones (logistic regression model)

In multivariate logistic regression models (also adjusted for age, sex, UI, BMI and smoking status), individuals with ferritin levels <30 μg/l were more likely to present hypothyroxinemia, and hypotriiodothyroninemia than the reference category with ferritin levels ≥30 µg/L. This association remained significant, considering a definition for these abnormalities as levels of FT4 and FT3 below p5 of the study population: OR 1.5 [IC95% 1.1–2.2] p = 0.024, and 1.8 [IC95% 1.3–2.6] p = 0.001 respectively, and also considering a more stringent criteria (FT4 and FT3 < p2.5): OR 1.7 [IC95% 1.0–2.7] p = 0.044 and 2.0 [IC95% 1.2–3.3] p = 0.007 respectively. However, as in the linear model, no association was found between ID and higher TSH, neither ≥p95: 0.8 [IC95% 0.6–1.2] p = 0.348 nor ≥97.5 (≥5.8 µUI/mL) OR 0.9 [IC95% 0.5–1.5] p = 0.676 (Table 3).

**Table 3:** Prevalence (%) and adjusted Odd Ratios (OR) for presenting high TSH, low FT4 and low FT3 in the study. Individuals according to their Ferritin levels (<30 vs ≥30 µg/L).

		Total number	Number (%)	Adjusted OR	95% CI	p	Number (%)	Adjusted OR	95% CI	p
			**TSH ≥ 4.7 µUI/mL (p95)**				**TSH ≥ 5.8 µUI/mL (p97.5)**			
Ferritin (µg/L)	**≥30**	2769	135 (4.9)	1			68 (2.5)	1		
	**<30**	1077	57 (5.3)	0.8	(0.6–1.2)	0.348	28 (2.6)	0.9	(0.5–1.5)	0.676
			**FT4 < 12.0 pmol/L (p5)**				**FT4 < 11.5 pmol/L (p2.5)**			
Ferritin (µg/L)	**≥30**	2769	117 (4.2)	1			52 (1.9)	1		
	**<30**	1075	73 (6.8)	1.5	(1.1–2.2)	0.024	41 (3.8)	1.7	(1.0–2.7)	0.044
			**FT3 < 3.9 pmol/L (p5)**				**FT3 < 3.8 pmol/L (p2.5)**			
Ferritin (µg/L)	**≥30**	2768	111 (4.0)	1			52 (1.9)	1		
	**<30**	1074	76 (7.1)	1.8	(1.3–2.6)	0.001	41 (3.8)	2.0	(1.2–3.3)	0.007

Adjusted ORs were calculated by logistic regression adjusted to age, sex, UI, BMI and smoking status.

### Subgroup analyses (logistic regression model)

**Figure 2:**
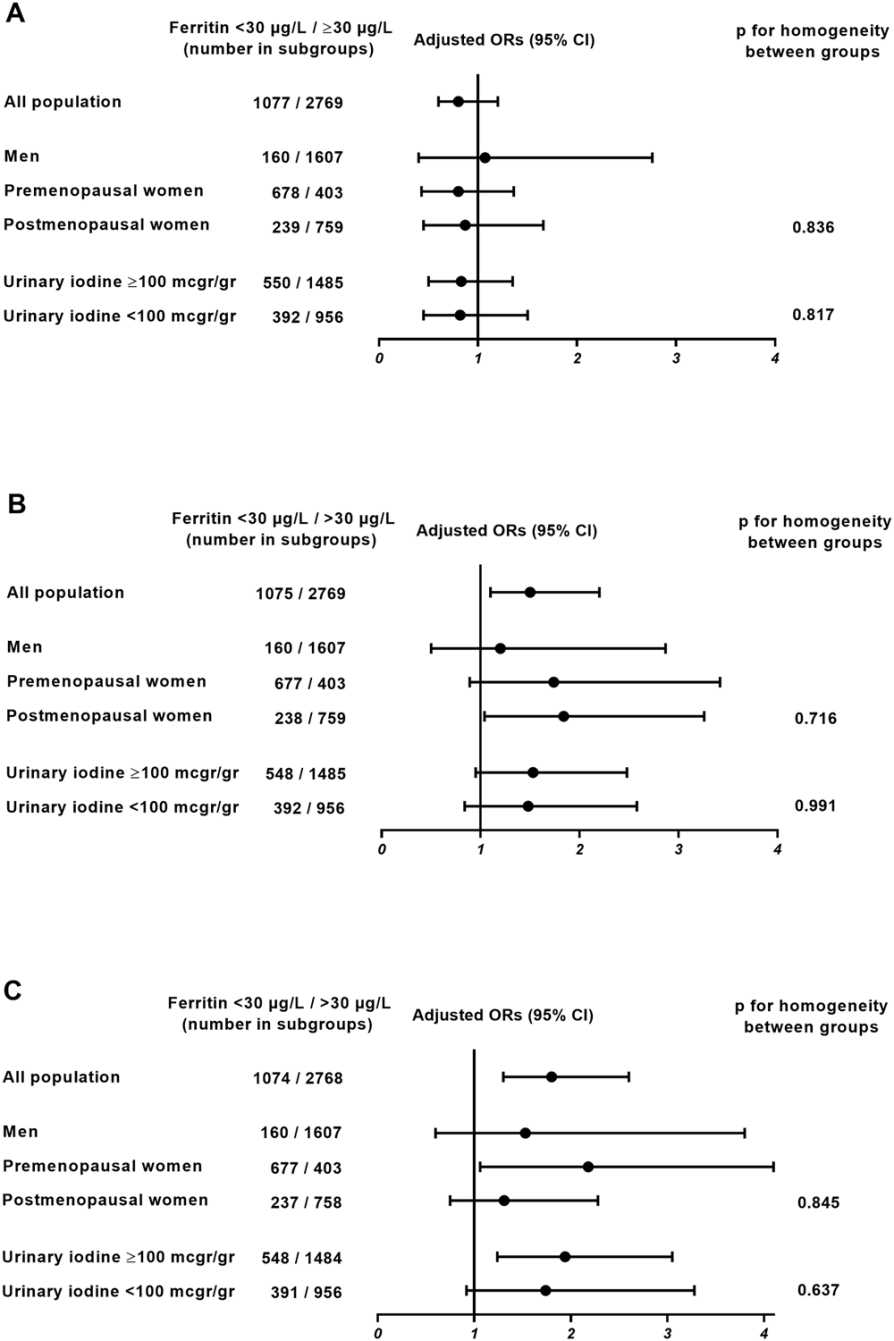
Adjusted Odd Ratios (ORs) for presenting high TSH (≥p95) (**A**), low FT4 (p < 5) (**B**), and low FT3 (<p5) (**C**), according to Ferritin levels in different population subgroups. Foot: Adjusted ORs were calculated by logistic regression adjusted to age, sex, UI, BMI and smoking status. Homogeneity of the ORs was tested with the Breslow-Day test.

Figure 2 shows the results of the logistic regression analyses in different population subgroups. As can be seen, although there were some small differences between groups, all the ORs tended toward the same direction, and no significant heterogeneity was seen in the results between men, pre-menopausal and post-menopausal women or according to the iodine nutrition status (iodine deficient vs. iodine replete). Subgroup analyses by using the more stringent criteria to define thyroid function abnormalities (low FT4 and FT3 < p2.5, and high TSH ≥ 97.5) were not performed due to the low number of subjects available for the analysis.

## Discussion

Our results confirm an association between ID and hypothyroxinemia and hypotriiodothyroninemia in a large sample of thyroid disease free subjects representative of the Spanish general adult population, in models controlled for possible confounding variables such as age, sex, BMI, smoking habit and UI.

These findings are in line with previous studies suggesting that ID may impair thyroid hormone production: ID has been shown to lower FT4 as well as FT3 in rats^6–8^, and small samples of humans with ID, with or without anemia, have been reported to have lower FT4 and FT3 levels than controls without ID^9,10^. Other previous studies have associated ID with the presence of goiter^18^, and iron supplementation in children with goiter and ID anemia has been shown to improve the efficacy of iodized oil administration, causing a reduction in glandular size^19,20^. ID has also been associated with changes in thyroid function in children and adolescents^11,13^, which improves after its correction^12^. More recently, ID has been identified as an independent risk factor for the development of hypothyroxinemia and thyroid dysfunction in pregnant women^14–17^. To our knowledge, this is the first epidemiological study reporting this association in a wide sample of the adult non-pregnant population, including adult men, as well as pre-menopausal and post-menopausal women.

Interestingly, the association between ID and the decreased levels of FT4 and FT3 found in our study is already significant for serum ferritin levels below 30 μg/l, which is in line with the level suggested by Mast *et al*.^21^. This threshold possibly corresponds to the so called “iron depletion state” defined by the WHO^22^ in which no hematological, cerebral or muscular alterations are observed, however an objective low iron storage is present in the organism. In fact, according to our results, lower ID thresholds (e.g. <15 μg/L) could understate the hypothyroxinemia and hypotriiodothyroninemia associated with ID.

ID may alter the synthesis of thyroid hormones by multiple mechanisms: ID induces ineffective erythropoiesis, thus reducing the transport of oxygen to the different tissues required for multiple enzymatic reactions^23^. ID has also been shown to increase *in-vitro* hepatic reverse triiodothyronine deiodination, suggesting that under conditions of ID, thyroid hormones tend to be metabolized by an inactivating route^24^. Also, ID may lower thyroperoxidase activity and therefore interfere with the synthesis of thyroid hormones^25^. Although these mechanisms described could be explanatory, our results suggest the implication of additional hypothalamic-pituitary mechanisms, given the lack of TSH response to the decrease in the levels of peripheral hormones. Accordingly, the experiments carried out by Tang *et al*. in iron deficient rats subjected to low temperatures, showed decreased levels of TSH, FT4 and FT3, which reverted to normal if stimulated with synthetic thyrotropin releasing hormone (THR), suggesting that ID may impair the hypothalamic secretion of THR^26^. In line with this observation, the study from Eftekhari in Iranian adolescents with ID, showed that despite increases in FT3 and FT4 concentrations, the TSH concentration was unaffected by iron supplementation^12^. Since no separate mechanism on its own fully explains the results found in our study, it is likely that a combination of mechanisms may be involved.

Our results are limited due to the cross sectional nature of the analyses, so we cannot infer causal mechanisms, however, the results may have clinical implications, especially in pre-menopausal women, where ID is more prevalent. In fact hypothyroxinemia is associated with numerous factors that complicate gestation, such as increased preterm birth or the development of gestational diabetes^27^ and the development of newborns such as language delay^28^ or lower intellectual coefficient^29^. Recently, the persistence of low T4 levels in children born to mothers with hypothyroxinemia and ID until the tenth postnatal day has also been demonstrated^30^. Thus, the implication of ID in the pathogenesis of these thyroid disorders in pregnancy could open a new perspective for its prevention and early intervention and requires further research. On the other hand, hypothyroxinemia and hypotriiodothyroninemia have not been sufficiently studied in other population groups, so that the existence of some harmful effects in the non-pregnant population cannot be discarded.

In conclusion, we report an association between ID and hypothyroxinemia and hypotriiodothyroninemia in the Spanish general adult population. Several hypothalamic-pituitary and peripheral mechanisms may be involved. Further prospective and experimental studies are needed to establish causal mechanisms and to expand knowledge in this field.

## Methods

The Di@bet.es Study is a national, cross sectional, population based survey that was conducted in 2009–10. A cluster sampling design was used to select participants to form a representative random sample of the Spanish population. In the first stage, 100 health centers or their equivalent were selected from all around the country, with a probability proportional to their population size, after which 100 individuals aged ≥18 years were randomly selected from each health center. Of the eligible adults, 55.8% attended for examination, of whom 9.9% were excluded (institutionalized, severe disease, pregnancy or recent delivery), giving a final sample of 5061 individuals. In 90% of this sample, thyroid function studies were performed^31^.

For the present analysis, we excluded all the subjects with a previous thyroid disease diagnosis, and/or taking interfering medications (levothyroxine, thionamides, amiodarone or lithium). Individuals with a positive TPOAbs test (≥50 IU/ml), or with very high (>20 mIU/L) or suppressed (<0.1 mIU/L) TSH levels, and/or with severe iodine deficiency (UI < 20 μg/g) were also excluded.

The research was carried out in accordance with the Declaration of Helsinki (2008) of the Word Medical Association. Written informed consent was obtained from all the participants. The study was approved by the Ethics and Clinical Investigation Committee of the Hospital Regional Universitario de Málaga (Malaga, Spain) in addition to other regional ethics and clinical investigation committees all over Spain.

### Variables and procedures

The participants were invited to attend a single examination visit at their health center. Information was collected using an interviewer administered structured questionnaire, followed by a physical examination and blood sampling.

Information on demographic characteristics and smoking status was obtained by questionnaire. Medical history and medications were also recorded. Menopause was considered in women who reported more than 12 months of amenorrhea without any other obvious pathological or physiological cause. Weight and height were measured and the body mass index was calculated by standardized methods. Blood samples were obtained in fasting conditions, were immediately centrifuged and the serum was frozen until analysis. A casual urinary sample (in the morning and in fasting state) was also collected and samples were frozen until analysis. Samples were managed and provided by the Hospital Regional Universitario de Málaga-IBIMA Biobank that also belongs to the Andalusian Public Health System Biobank, and the biorepository CIBERDEM (Instituto de Salud Carlos III) managed by IDIBAPS Biobank.

TSH, FT4, FT3 and TPOAb concentrations were analyzed using an electrochemiluminescence immunoassay (Modular Analytics E170, cobas e 602, Roche Diagnostics, Basel, Switzerland). The functional sensitivity of the TSH assay was 0.014 mIU/L. The intra-assay coefficients of variation were: TSH, 1.5–1.2%; FT4 1.8–1.6%; FT3 1.3–2.0% and TPOAb 4.8–2.8%. The inter-assay coefficients of variation for the low and high levels of serum TSH, FT4, FT3 and TPOAb quality control materials were 3.5 and 2.7%, 4.17 and 2.64%, 3.78 and 2.21%, and 8.5 and 5.2%, respectively. All samples were analyzed at the laboratory of Biochemistry of the Hospital Regional Universitario de Malaga, which attends a population of 487,857 people from the city of Malaga.

UI was analyzed using the modified method of Benotti and Benotti^32^. The intra- and inter-assay coefficients of variation of UI assay were 2.01% and 4.53%, respectively.

Ferritin levels were analysed by immunochemiluminescence in the Architect I2000 analyzer (Abbott Laboratories SA, Madrid, Spain). The intra- coefficients of variation of the assay was 5.6% and the functional sensitivity 0.1055 ng/mL. ID was defined as ferritin levels <30 µg/L as proposed by Mast *et al*.^21^.

### Statistical analysis

To test the association between ferritin and thyroid hormones, we calculated the estimated mean levels of TSH, FT4 and FT3 in different ferritin categories in general linear models adjusted for possible confounders such us age, sex, UI, BMI and smoking status. Because they were nonnormally distributed, serum TSH values were log-transformed.

We also constructed logistic regression models to calculate the odds ratios for having low FT4 levels (<p5 and <p2.5) low FT3 levels (<p5 and <p2.5) and high TSH (>p95 and >p97.5), according to the ferritin levels (dichotomized as <30 vs ≥30 µg/L) adjusted for these same variables (age, sex, UI, BMI and smoking status). We used the Breslow-Day test to test for homogeneity of the odds ratios according to various clinical categories (men vs. pre-menopausal vs. post-menopausal women, iodine deficient vs. iodine replete).

Reported p values were based on two-sided tests with statistical significance set at 0.05.

### Data availability

The datasets generated during and/or analyzed during the current study are available from the corresponding author on reasonable request.
